# Bilateral Lateral Tarsal Artery Bypass and Free Flap Reconstruction of an Infected Gangrenous Heel in Chronic Limb-Threatening Ischemia: A Case Report of Three-Year Functional Limb Salvage

**DOI:** 10.70352/scrj.cr.26-0684

**Published:** 2026-10-31

**Authors:** Noriyuki Yokoyama, Shinsuke Kikuchi, Hirofumi Jinno, Takayuki Uramoto, Kazuki Takahashi, Keisuke Kamada, Takamitsu Tatsukawa, Uran Moriyama, Yuri Yoshida, Ranko Hinooka, Takanori Fujiki, Nobuyoshi Azuma

**Affiliations:** 1 Department of Vascular Surgery, Asahikawa Medical University, Asahikawa, Hokkaido, Japan; 2 Department of Chronic Limb-Threatening Ischemia Research, Asahikawa Medical University, Asahikawa, Hokkaido, Japan; 3 Department of Plastic and Reconstructive Surgery, Asahikawa Medical University, Asahikawa, Hokkaido, Japan; 4 Department of Nursing, Asahikawa Medical University Hospital, Asahikawa, Hokkaido, Japan; 5 Dialysis Center, Japanese Red Cross Shimizu Hospital, Kamikawa-gun, Hokkaido, Japan

**Keywords:** chronic limb-threatening ischemia, lateral tarsal artery, distal bypass, heel gangrene, free latissimus dorsi flap, functional limb salvage

## Abstract

**INTRODUCTION:**

Distal bypass for chronic limb-threatening ischemia (CLTI) is technically demanding when the conventional pedal arteries are unavailable, and revascularization alone seldom heals an extensive heel defect.

**CASE PRESENTATION:**

A man in his 60s receiving maintenance hemodialysis presented with bilateral CLTI and gangrene of the left heel after failed endovascular therapy, and bilateral major amputations had been considered before referral. Both limbs were classified as Wound, Ischemia, and foot Infection clinical stage 4. Diffuse calcification of the infrapopliteal and pedal arteries left the lateral tarsal artery (LTA) as the only suitable distal target, and bilateral superficial femoral artery-to-LTA bypasses were performed with in situ great saphenous vein grafts under nerve block anesthesia. Because repeated debridement left the calcaneus exposed, a free latissimus dorsi musculocutaneous flap anastomosed to the left bypass graft was transferred and covered with a split-thickness skin graft. The wounds healed after minor amputations, and the patient regained independent ambulation. An ulcer subsequently developed on the weight-bearing surface of the transferred flap and never healed despite pressure-relieving insoles and additional inflow angioplasty; nevertheless, the flap remained viable, both grafts remained patent, and ambulatory function was preserved until the patient died of an unrelated cause 3 years after revascularization.

**CONCLUSIONS:**

The LTA can serve as a distal bypass target when the conventional pedal arteries are unavailable, and, when combined with free flap reconstruction, it may achieve long-term functional limb salvage despite extensive heel gangrene. The reconstructed weight-bearing heel nevertheless remains prone to ulceration that may not heal, so preventive off-loading and long-term surveillance are essential.

## Abbreviations

ABI
: ankle–brachial index
CLTI
: chronic limb-threatening ischemia
DPA
: dorsalis pedis artery
EVT
: endovascular therapy
GSV
: great saphenous vein
LD
: latissimus dorsi
LTA
: lateral tarsal artery
PTA
: posterior tibial artery
SFA
: superficial femoral artery
SPP
: skin perfusion pressure
WIfI
: Wound, Ischemia, and foot Infection

## INTRODUCTION

CLTI is the most advanced stage of peripheral arterial disease and carries a high risk of major amputation, cardiovascular events, and death.^1,2)^ Patients with diabetes mellitus and end-stage renal disease frequently present with diffuse infrapopliteal and pedal artery occlusive disease accompanied by severe medial arterial calcification, in which autogenous vein bypass remains the preferred strategy when durable EVT is unlikely to be achieved.^1,2)^ The DPA or the plantar arteries are usually selected as distal target vessels,^3)^ whereas the LTA is rarely used because of its small caliber and anatomical variability, and reports describing LTA bypass remain limited.^4,5)^

Even after successful revascularization, extensive heel defects remain difficult to treat, because the heel is a weight-bearing region that requires durable soft-tissue coverage to restore ambulation.^6)^ Combined revascularization and free flap reconstruction have emerged as an effective multidisciplinary strategy for functional limb salvage,^7,8)^ although ulcer recurrence remains common during long-term follow-up despite successful flap survival.^8)^

We report a case of bilateral CLTI with severe pedal artery calcification in which the LTA was the only feasible distal bypass target and revascularization alone could not heal an extensive infected heel defect. Bilateral SFA-to-LTA bypasses combined with free LD flap reconstruction achieved wound healing and preserved ambulatory function until the patient’s death 3 years after revascularization.

## CASE PRESENTATION

A man in his 60s receiving maintenance hemodialysis was referred to our institution for bilateral CLTI. He had undergone EVT at a previous hospital, including stent implantation in the left SFA, but the infrapopliteal and pedal lesions were heavily calcified and unsuitable for further EVT, and bilateral major amputations had been considered before referral. His medical history included hypertension, diabetes mellitus, end-stage renal disease, permanent pacemaker implantation, and smoking (BMI, 25.1 kg/m²). He had been ambulatory without assistance before the onset of CLTI but was unable to walk at presentation because of ischemic pain and bilateral foot wounds.

The popliteal artery pulses were palpable bilaterally, whereas the DPA and PTA pulses were absent. Gangrene of the left first and second toes was accompanied by an extensive infected gangrenous ulcer of the left heel, and gangrene of the first toe was present on the right side (**Fig. 1A**–**1C**). Laboratory tests showed a white blood cell count of 15770/μL, a C-reactive protein level of 11.3 mg/dL, an albumin level of 2.7 g/dL, and a creatinine level of 6.21 mg/dL. A wound culture obtained from the left heel on admission yielded Escherichia coli, Leclercia adecarboxylata, and Candida parapsilosis. Empirical intravenous meropenem (0.5 g once daily) and vancomycin (0.5 g on hemodialysis days) were initiated immediately after culture sampling, together with wound irrigation and local treatment with cadexomer iodine ointment. Osteomyelitis was not specifically assessed. The left ventricular ejection fraction was 54% without regional wall motion abnormality.

**Fig. 1:**
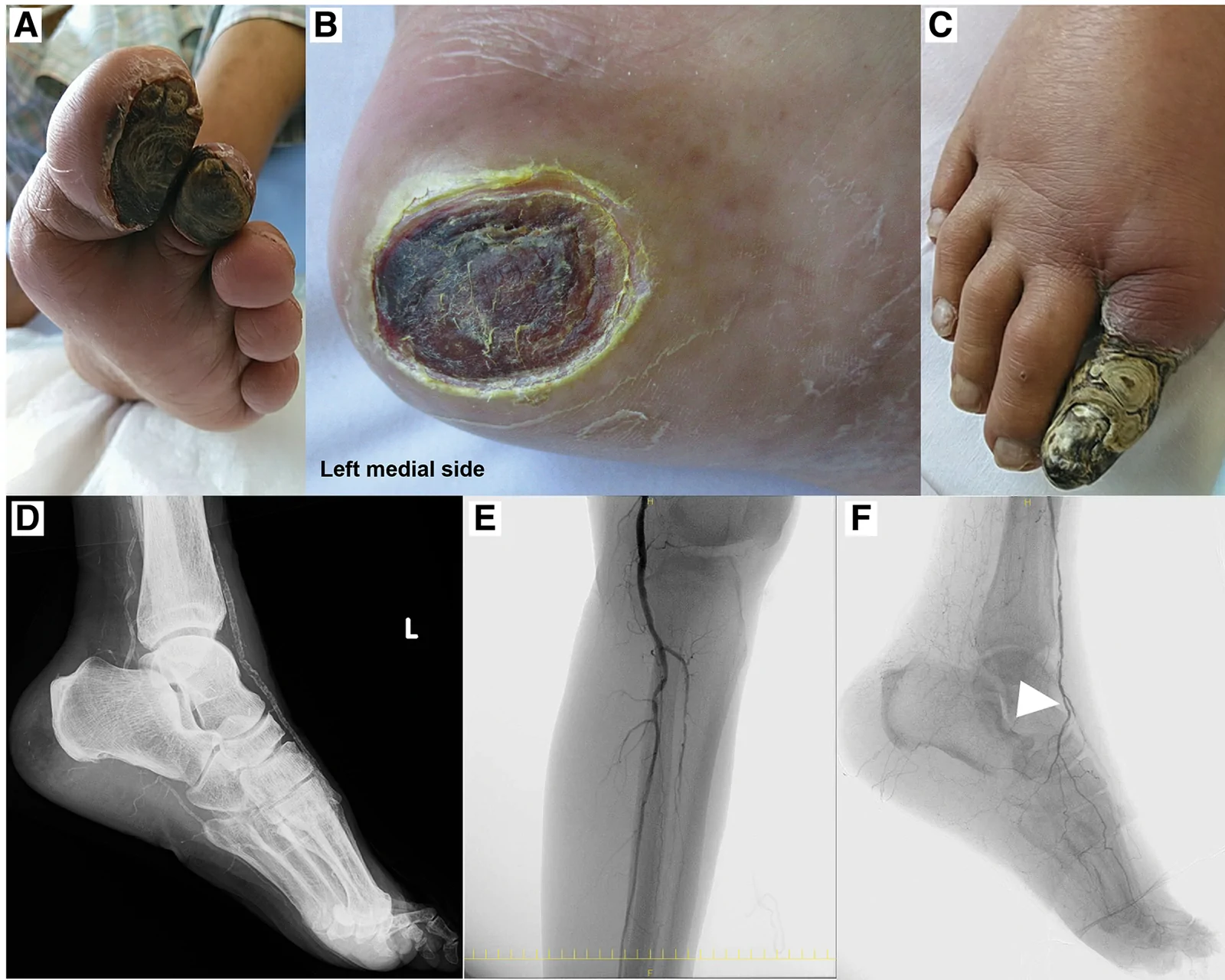
Preoperative findings. (**A**) Gangrene of the first and second toes of the left foot. (**B**) Extensive infected gangrenous ulcer of the left heel with full-thickness tissue loss (left medial side). (**C**) Gangrene of the first toe of the right foot. (**D**) Lateral radiograph of the left foot showing severe medial calcification of the pedal arteries. (**E**) Preoperative DSA of the left lower leg showing segmental occlusion of the anterior tibial artery and complete occlusion of the PTA. (**F**) Preoperative DSA of the left foot showing occlusion of the DPA distal to the origin of a patent LTA; the arrowhead indicates the planned distal anastomotic site. DPA, dorsalis pedis artery; LTA, lateral tarsal artery; PTA, posterior tibial artery

The ABI was 0.77 on the right and 0.99 on the left. Because medial arterial calcification renders the ABI unreliable, ischemia was graded using SPP measured in the region of each wound: 51 mmHg at the dorsum and 15 mmHg at the plantar surface on the right, and 43 and 37 mmHg on the left, corresponding to ischemia grade 3 and grade 2, respectively. Both feet showed local infection without systemic inflammatory response syndrome (foot infection grade 2). Both limbs were therefore classified as WIfI clinical stage 4 (right, W2 I3 fI2; left, W3 I2 fI2, reflecting full-thickness tissue loss of the weight-bearing heel).

Radiography demonstrated severe medial calcification extending to the pedal arteries (**Fig. 1D**). DSA showed segmental occlusion of the left anterior tibial artery, complete occlusion of the PTA, and a patent proximal peroneal artery (**Fig. 1E**). The DPA was occluded distal to the origin of the LTA, whereas the LTA was patent and reconstituted the pedal circulation, leaving the LTA as the only suitable distal target vessel (Global Limb Anatomic Staging System stage III) (**Fig. 1F**). Similar severe pedal artery disease was present in the contralateral limb. Despite palpable popliteal pulses, bilateral popliteal artery calcification precluded their use for proximal anastomosis. Given the prior long-segment stenting of the left SFA and diffuse calcification with focal stenosis of the right SFA, suitable proximal SFA segments were selected as inflow sites.

Because the left heel gangrene was associated with progressive infection, urgent revascularization was prioritized over coronary intervention despite calcified 3-vessel coronary artery disease. Surgery was performed under combined femoral and sciatic nerve block anesthesia,^9)^ and the ipsilateral GSV was used in an in situ fashion in both limbs. Preoperative radiography showed no evident calcification at the planned LTA anastomotic segment. Duplex ultrasonography was used to identify the DPA and LTA, and their courses were marked on the skin. After intraoperative angiographic confirmation, the DPA was exposed through a dorsal foot incision and dissected distally to identify the LTA for anastomosis (**Fig. 2A**, **2B**). The distal GSV was mobilized through separate skin incisions, which were staggered relative to the LTA incision to prevent necrosis of the intervening skin (**Fig. 2B**). After destruction of the venous valves with a valvulotome (ELVH-002-C; LeMaitre Vascular, Burlington, MA, USA), the proximal anastomosis was completed with 6-0 polypropylene sutures and the distal anastomosis with 8-0 polypropylene sutures. Completion angiography, performed by injecting contrast medium with a syringe through a 2-mm cannula inserted into a GSV tributary in the lower leg (**Fig. 2C**), demonstrated satisfactory patency and runoff through the LTA (**Fig. 3A**), and transit-time flow measurement (VeriQ; Medistim ASA, Oslo, Norway) showed a graft flow of 30 mL/min. Extensive debridement of the infected heel wound and amputation of the first and second toes at the metatarsophalangeal joints were performed simultaneously.

**Fig. 2:**
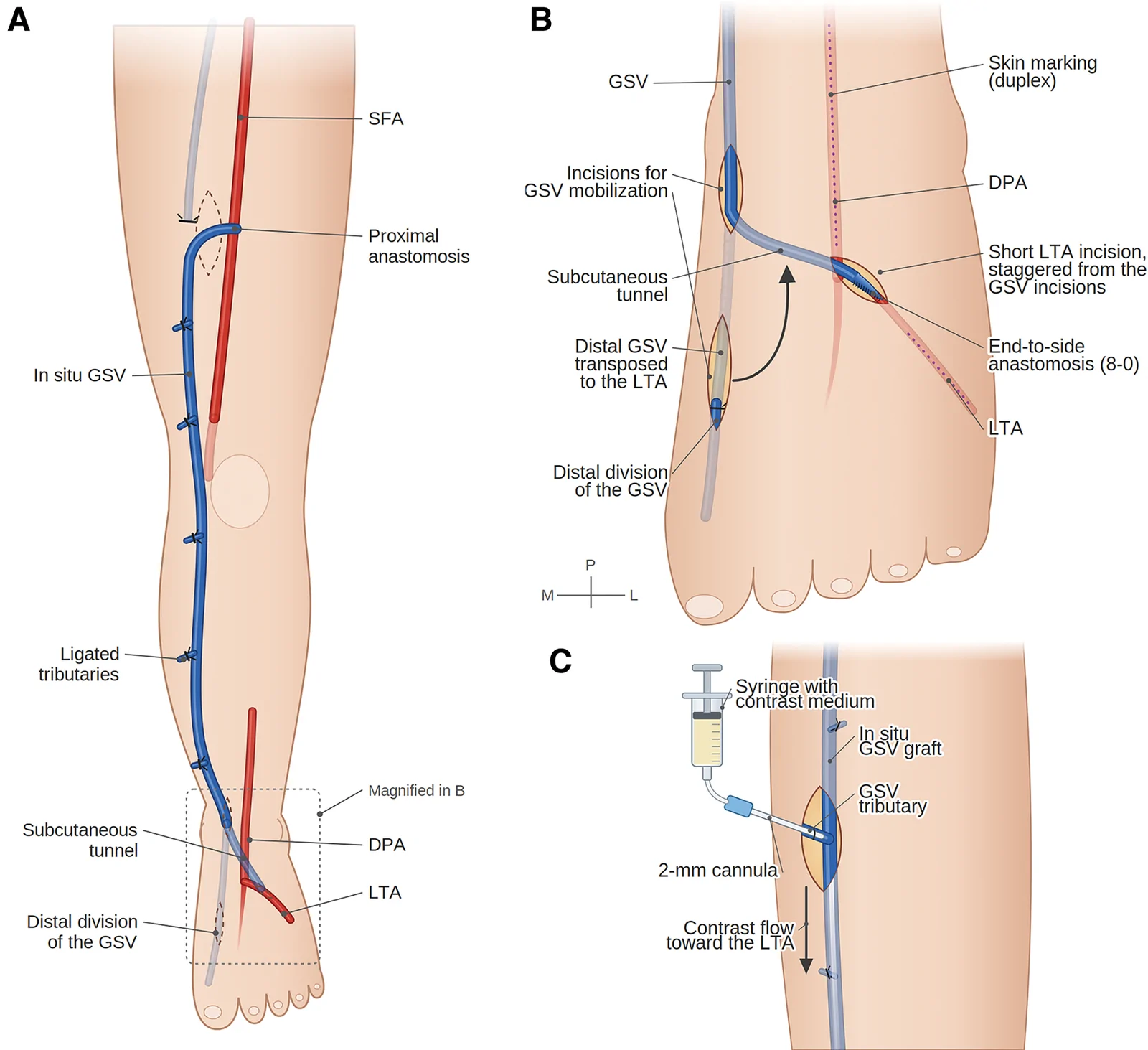
Schematic illustrations of the left SFA-to-LTA bypass using an in situ GSV graft. (**A**) Overview of the procedure: proximal anastomosis between the SFA and the GSV through a thigh incision; the in situ GSV with ligated tributaries; and the subcutaneous route of the distally divided GSV across the dorsum of the foot to the LTA. The boxed area is magnified in (**B**). (**B**) Magnified view of the dorsum of the foot. The distal GSV is mobilized through separate skin incisions, divided distally, and tunneled subcutaneously to a short incision over the LTA, where the LTA is exposed by distal dissection from the DPA and the end-to-side distal anastomosis is performed. The LTA incision is staggered relative to the GSV incisions to avoid necrosis of the intervening skin. (**C**) Schematic illustration of completion angiography: contrast medium is injected with a syringe through a 2-mm cannula inserted into a GSV tributary in the lower leg and flows distally through the graft toward the LTA. The resulting completion angiogram is shown in **Fig. 3A**. DPA, dorsalis pedis artery; GSV, great saphenous vein; LTA, lateral tarsal artery; SFA, superficial femoral artery

**Fig. 3:**
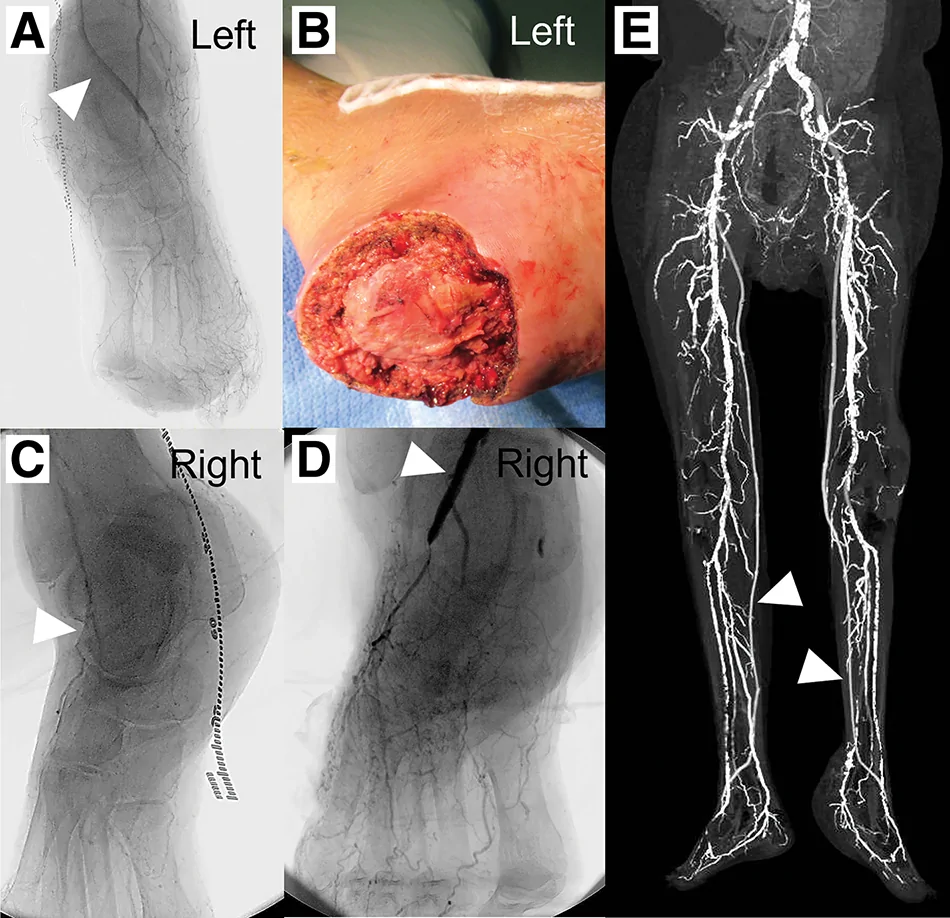
Revascularization and residual heel defect. (**A**) Completion angiography after left SFA-to-LTA bypass, showing a patent in situ GSV graft and satisfactory runoff through the LTA (arrowhead, distal anastomosis). (**B**) Left heel wound after repeated debridement, showing extensive soft-tissue loss with calcaneal exposure. (**C**) Preoperative angiography of the right foot demonstrating a patent LTA; the arrowhead indicates the planned distal anastomotic site. (**D**) Completion angiography after right SFA-to-LTA bypass, showing a patent graft with the distal anastomosis at the LTA (arrowhead). (**E**) Early postoperative 3D CTA demonstrating patency of both bypass grafts (arrowheads). GSV, great saphenous vein; LTA, lateral tarsal artery; SFA, superficial femoral artery

Repeated debridement converted the left heel wound into a full-thickness defect with calcaneal exposure (**Fig. 3B**). Vancomycin was subsequently discontinued, and a follow-up wound culture became negative on POD 9 after the left bypass. Meropenem was continued because the heel wound remained open. Percutaneous coronary intervention for coronary artery disease was performed on POD 9 once the local infection had stabilized, and right SFA-to-LTA bypass was performed 14 days after the initial operation using the same strategy (graft flow, 28 mL/min; **Fig. 3C**, **3D**). CTA confirmed patency of both grafts (**Fig. 3E**). Postoperative SPP could not be measured, mainly because extensive tissue loss from heel and forefoot necrosis on the left and forefoot necrosis on the right precluded reliable measurements in the wound regions, and also because prolonged bed rest was required for wound management, free flap reconstruction, and subsequent skin grafting. Because the exposed calcaneus made spontaneous wound healing unlikely, free LD musculocutaneous flap reconstruction was planned.

43 days after the initial bypass, an 8 × 20 cm LD musculocutaneous flap was transferred in collaboration with the plastic surgery team. At the time of flap reconstruction, linezolid (600 mg once daily) was added to meropenem and continued until 50 days after the initial bypass. The bypass graft and the posterior tibial vein were exposed through a medial ankle incision, and necrotic bone was removed until viable cancellous bone was exposed (**Fig. 4A**–**4C**). The thoracodorsal artery was anastomosed in an end-to-side fashion to the bypass graft with interrupted 9-0 nylon sutures, and the thoracodorsal vein was anastomosed end to end to the posterior tibial vein. Indocyanine green angiography confirmed satisfactory flap perfusion, and a split-thickness skin graft harvested from the right thigh was applied over the exposed muscle surface (**Fig. 4D**).

**Fig. 4:**
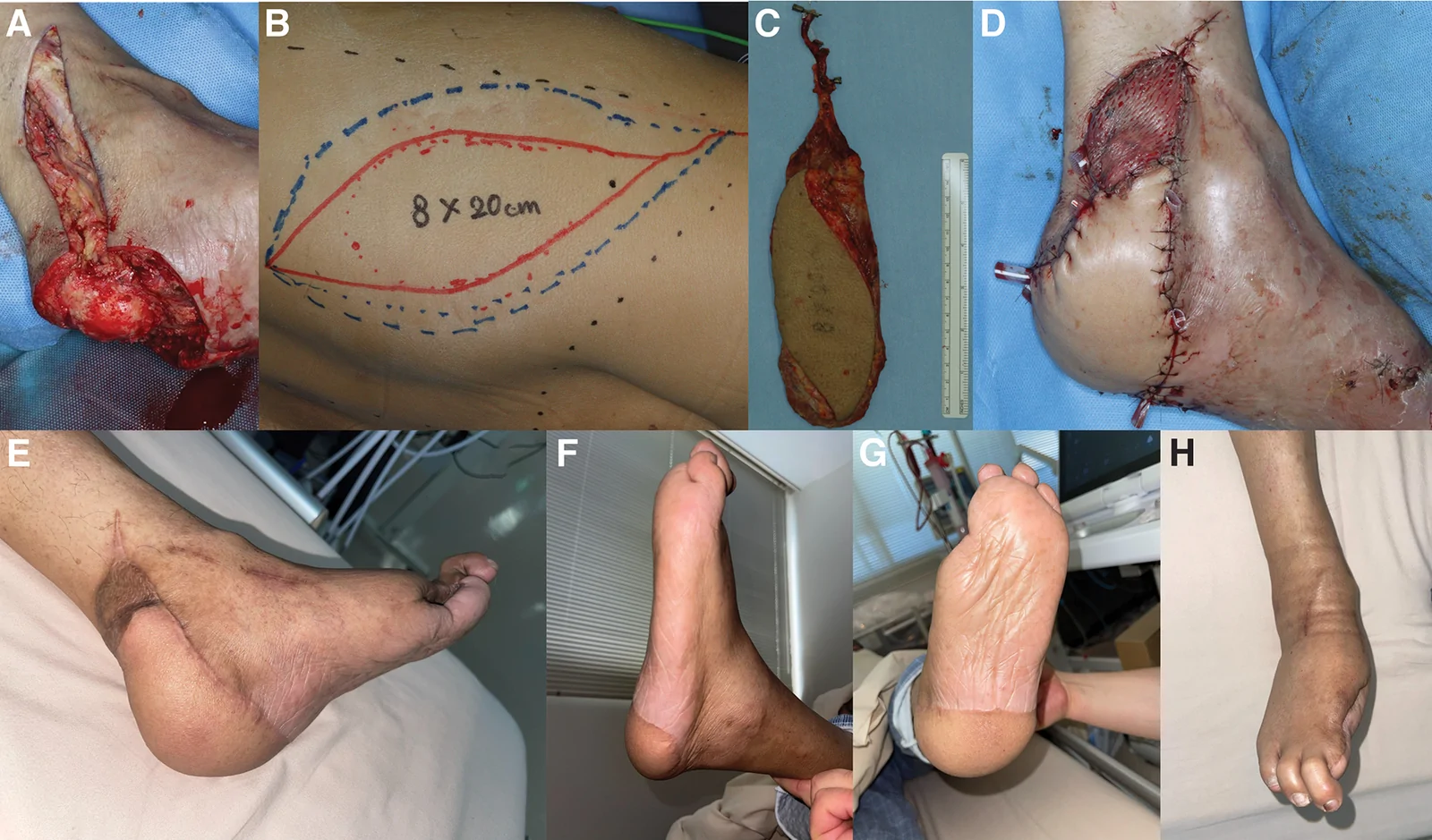
Free LD flap reconstruction and wound healing. (**A**) Exposure of the bypass graft and the posterior tibial vein through a medial ankle incision after debridement of the heel wound. (**B**) Design of the 8 × 20 cm LD musculocutaneous flap on the back. (**C**) Harvested flap with the thoracodorsal pedicle. (**D**) Appearance immediately after flap inset with split-thickness skin grafting. (**E**–**H**) Completely healed left heel (**E**–**G**) and right forefoot stump (**H**) 98 days after the initial bypass. LD, latissimus dorsi

Strict off-loading of the heel was maintained, and meropenem was discontinued 16 days after flap transfer. Duplex ultrasonography showed a graft flow of 52 mL/min on the right and 153 mL/min on the left. Partial weight-bearing was introduced 41 days after flap transfer and progressed to full weight-bearing under the supervision of the rehabilitation team. Both forefoot wounds healed after bilateral partial first- and second-ray amputation, which was required on the right side because deep infection extended beyond the gangrenous first toe. At 98 days after the initial bypass, complete wound healing had been achieved (**Fig. 4E**–**4H**), and the patient had regained ambulatory function with a walker.

He subsequently regained independent outdoor mobility (**Fig. 5A**). However, 480 days after the initial bypass, a small ulcer developed on the weight-bearing surface of the transferred flap because of repetitive loading (**Fig. 5B**), although DSA demonstrated sustained graft patency (**Fig. 5C**). Custom-made pressure-relieving insoles were provided in addition to local wound care, but the ulcer did not improve, and balloon angioplasty of a pre-existing stenosis of the left SFA was performed 605 days after the initial bypass to improve inflow. The ulcer never healed and enlarged slowly thereafter, although the flap itself remained viable without total breakdown and both grafts remained patent (**Fig. 5D**–**5G**). The patient maintained ambulatory function until he died of subarachnoid hemorrhage 1097 days after the initial bypass, with both limbs preserved.

**Fig. 5:**
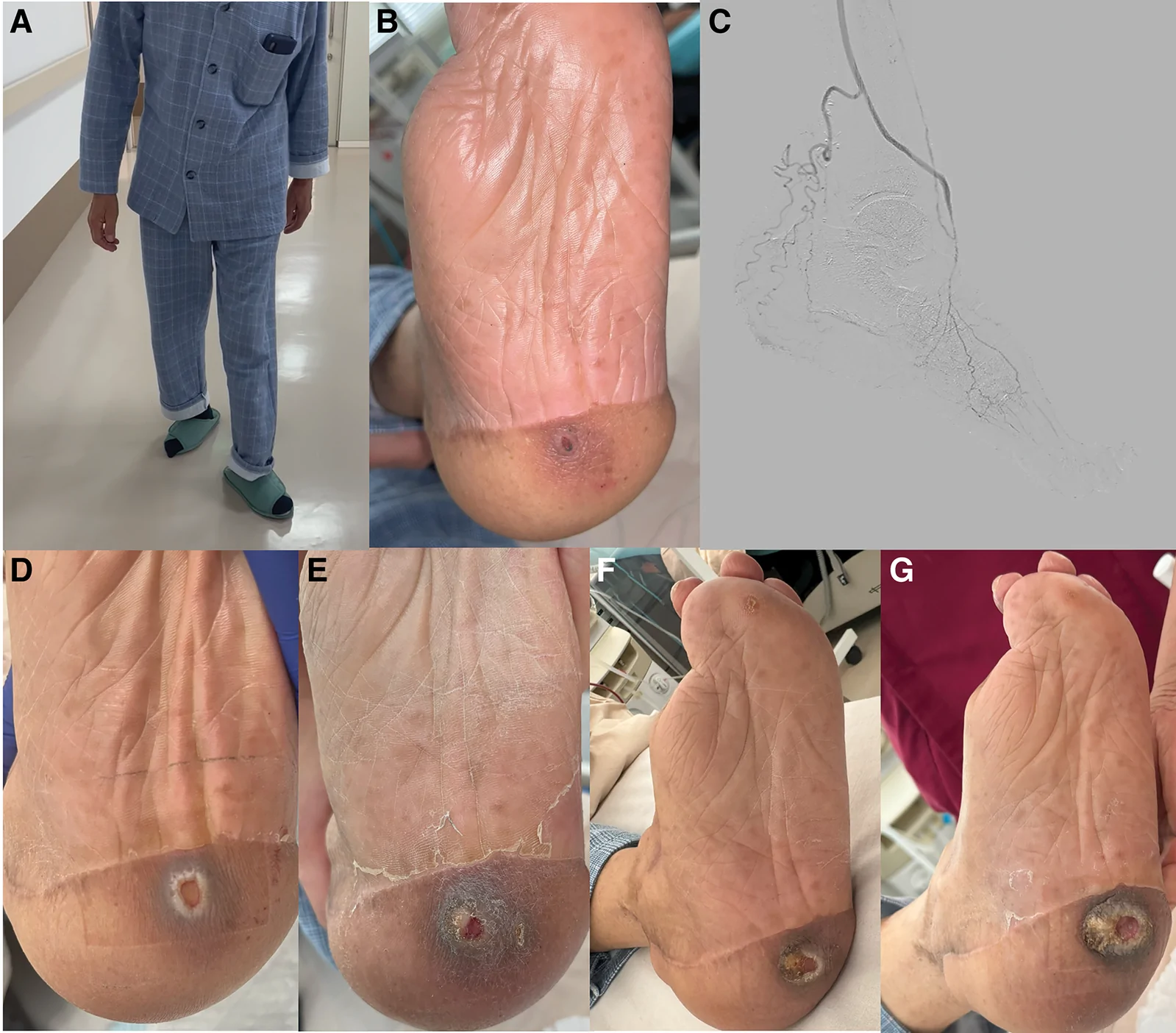
Long-term follow-up. (**A**) Independent ambulation without an assistive device at an outpatient visit after multidisciplinary treatment and rehabilitation. (**B**) Ulcer that developed on the weight-bearing surface of the transferred flap. (**C**) DSA at the time of ulcer recurrence demonstrating sustained patency of the bypass graft. (**D**–**G**) Serial photographs of the recurrent heel ulcer obtained 472, 563, 626, and 731 days after flap transfer (515, 606, 669, and 774 days after the initial bypass), showing gradual progression of the ulcer despite sustained graft patency and flap viability.

## DISCUSSION

This case illustrates 3 points relevant to limb salvage in patients on maintenance hemodialysis: the diagnostic pitfall of relying on the ABI, the feasibility of the LTA as a distal bypass target when the conventional pedal arteries are unavailable, and the long-term vulnerability of a flap-reconstructed weight-bearing heel. Medial arterial calcification limits the diagnostic value of the ABI. In the present patient, the ABI underestimated the severity of ischemia in both limbs, corresponding to WIfI ischemia grade 1 on the right and grade 0 on the left, whereas SPP measurements disclosed grade 3 and grade 2 ischemia, respectively. SPP-based WIfI staging therefore determined both the urgency of revascularization and the choice of the surgical strategy. The optimal revascularization strategy should be individualized according to patient risk, anatomical complexity, life expectancy, and functional goals; furthermore, contemporary evidence from the BEST-CLI, SPINACH, and BASIL trials supports complementary roles for open bypass surgery and EVT.^10–12)^ In the SPINACH study, in which a large proportion of the Japanese patients were receiving maintenance hemodialysis, bypass surgery was advantageous in subgroups with extensive tissue loss and poor infrapopliteal runoff,^11)^ a profile closely resembling that of our patient.

The LTA is an uncommon bypass target because of its small caliber and anatomical variability, and successful distal bypass may depend on selecting the best available runoff vessel rather than adhering to a predetermined anatomical target. Because the vessel lies deep to the extensor digitorum brevis, preoperative duplex ultrasonography, together with intraoperative angiography and Doppler examination, is helpful for localizing and exposing it safely.^4)^ Bypass to the plantar and tarsal arteries provides acceptable patency and limb salvage rates, although early graft failure occurs more frequently than after DPA bypass.^5)^ In the present case, bilateral LTA bypasses remained patent throughout follow-up despite advanced pedal artery disease.

Direct revascularization of the wound-related angiosome has been associated with improved wound healing.^13,14)^ In a distal bypass series from our institution, wound healing was slower after indirect than after direct revascularization, and this difference was most pronounced in patients with end-stage renal disease; heel ulceration was likewise an independent negative predictor of healing.^13)^ Direct arterial flow to the wound-related angiosome should therefore be pursued whenever possible in patients receiving maintenance hemodialysis, in whom the pedal collateral network is frequently impaired by severe medial calcification. In our patient, the heel ulcer belonged to the posterior tibial angiosome, but the PTA was occluded without any usable distal target, so that only indirect revascularization through the LTA was feasible. Wound healing was nonetheless achieved, probably because the free flap anastomosed directly to the bypass graft brought its own blood supply to the defect, which may partly compensate for the disadvantage of indirect revascularization in such patients.

Restoration of arterial blood flow alone is insufficient for extensive heel defects, because the weight-bearing heel also requires stable soft-tissue coverage.^6)^ Combined revascularization and free tissue transfer have been used at our institution for extensive tissue loss since the era of distal venous arterialization,^15)^ and a recent report showed that a free LD musculocutaneous flap anastomosed to the revascularization graft maintained limb preservation even after graft inflow had deteriorated.^16)^ This combined approach provides durable limb salvage despite frequent wound complications.^7,17)^ Early soft-tissue reconstruction is therefore 1 option within the overall treatment strategy, rather than only a salvage procedure after failed wound healing. Whether free flap coverage is genuinely advantageous for the heel nevertheless remains open to question, because ulcer recurrence in weight-bearing regions is common even when perfusion is adequate,^8)^ as in our patient. If flap coverage is undertaken, measures to prevent ulceration of the reconstructed surface should be planned from the outset.

Several factors may account for this recurrence. Ulceration of the reconstructed surface is a recognized late complication after heel coverage with either muscle or fasciocutaneous flaps,^18)^ because neither the flap nor the overlying skin graft reproduces the fibrous septal architecture of the native heel pad, and since the reconstructed surface is insensate, it tolerates repetitive pressure and shear poorly. In addition, the transferred LD muscle is denervated, and a prospective randomized study using histology and MRI showed that denervated LD muscle undergoes progressive myofiber atrophy with fatty replacement, such that flap bulk is maintained largely by adipose tissue rather than contractile tissue.^19)^ Once an ulcer had developed over the reconstructed heel in our patient, it failed to heal despite pressure-relieving insoles and improvement of graft inflow. Whether this reflects the altered composition of the denervated flap, the absence of protective sensation, or simply persistent mechanical overload cannot be determined from a single case, and comparative data on the healing capacity of an ulcerated flap over the heel are lacking. Prevention therefore appears more important than treatment, and lifelong pressure redistribution using custom-made insoles and therapeutic footwear, together with regular foot surveillance, should be planned before weight-bearing is resumed.^20)^ Although the persistent ulcer represents a limitation of flap reconstruction of the weight-bearing heel, the flap itself remained viable and the patient retained independent ambulation until his death 3 years after the initial bypass, so that the overall goal of functional limb preservation was met.

Current management of CLTI has shifted from simple limb salvage toward functional limb salvage, emphasizing mobility and QOL rather than avoidance of major amputation alone.^21,22)^ Bilateral SFA-to-LTA bypasses combined with free LD flap reconstruction have rarely been reported, and although limited to a single case, this experience suggests that individualized distal bypass planning combined with microsurgical reconstruction and structured rehabilitation may achieve long-term functional limb salvage even in patients with severe pedal artery disease and extensive heel defects.

## CONCLUSIONS

Bilateral SFA-to-LTA bypasses combined with free LD flap reconstruction achieved long-term functional limb salvage in a patient with bilateral CLTI, severe pedal artery calcification, and an extensive heel defect. The LTA may serve as an alternative distal bypass target when the conventional pedal arteries are unavailable. Soft-tissue reconstruction of the heel, in contrast, remains problematic: even with adequate perfusion, the reconstructed weight-bearing surface is prone to ulceration that may not heal once it develops. Preventive off-loading with custom-made footwear and long-term surveillance should therefore be regarded as an integral part of the treatment strategy rather than an afterthought.

## DECLARATIONS

### Use of artificial intelligence

A generative artificial intelligence tool was used for English language editing of this manuscript. The authors reviewed and verified all content and took full responsibility for the manuscript.

### Availability of data and materials

The datasets supporting the conclusions of this article are included within the article.
